# NMDAR-CaMKII Pathway as a Central Regulator of Aggressiveness: Evidence from Transcriptomic and Metabolomic Analysis in Swimming Crabs *Portunus trituberculatus*

**DOI:** 10.3390/ijms252312560

**Published:** 2024-11-22

**Authors:** Qihang Liang, Dapeng Liu, Boshan Zhu, Fang Wang

**Affiliations:** 1 Key Laboratory of Mariculture, Ministry of Education, Ocean University of China, Qingdao 266003, China; lqh@stu.ouc.edu.cn (Q.L.); ldp@ouc.edu.cn (D.L.); zhuboshan@stu.ouc.edu.cn (B.Z.); 2 Function Laboratory for Marine Fisheries Science and Food Production Processes, Laoshan Laboratory, Qingdao 266237, China

**Keywords:** aggressiveness, NMDAR-CaMKII pathway, transcriptomic, metabolomic, neural energy state, *Portunus trituberculatus*

## Abstract

Aggressiveness is one of the personality traits of crustaceans, playing a crucial role in their growth, life history, and adaptability by influencing resource acquisition. However, the neuroregulatory mechanisms of aggressiveness in crustaceans remain poorly understood. The thoracic ganglion offers valuable insights into complementary aspects of aggression control. This study identified the aggressiveness of swimming crabs *Portunus trituberculatus*, conducted transcriptomic and metabolomic analyses of the thoracic ganglia, and confirmed the neural regulatory effects on aggressiveness. Behavioral analyses showed that highly aggressive individuals exhibited increased frequency and duration of chela extension, more frequent attacks, approaches and retreats, as well as extended movement distances. Omics analysis revealed 11 key candidate genes and three metabolites associated with aggressiveness, which were primarily enriched in pathways related to energy metabolism and neurodegeneration. Injection of an NMDAR activator significantly decreased aggressiveness in highly aggressive crabs, accompanied by a significant increase in NMDAR protein fluorescence intensity and downregulation of *NR2B*, *CaMKII*, and *CREB* genes. Conversely, when lowly aggressive crabs were injected with an NMDAR inhibitor, they showed increased aggressiveness alongside significantly decreased NMDAR protein fluorescence intensity, upregulated *NR2B* expression, and downregulated *CaMKII* and *CREB* genes. These results suggest that NMDAR within the thoracic ganglia serves as a key receptor in modulating aggressiveness in *P. trituberculatus*, potentially by influencing neural energy state via the NMDAR-CaMKII pathway, which in turn affects oxidative phosphorylation, cAMP, and FoxO pathways.

## 1. Introduction

Aggressiveness is a highly conserved behavior across animal evolution and is essential for survival, reproduction, and resource acquisition [1,2]. It is also a predictor of social dominance [3,4]. The level of aggressiveness displayed by individuals reflects their competitive abilities. For instance, highly aggressive individuals often exhibit competitive and resource-monopolizing behaviors, whereas less aggressive individuals tend to avoid direct competition and other costly social interactions [5,6]. Aggressiveness is a complex quantitative trait regulated by numerous genes [7]. Studies in zebrafish (*Danio rerio*) have identified 12 gene modules significantly correlated with the aggression duration [8]. Several gene families associated with aggressive behavior include cytochrome P450, components of electron transport chains, voltage-gated potassium channels, and genes involved in catabolic processes, G-protein-coupled receptor signaling, and core cellular metabolic pathways [9]. Extensive gene expression patterns can be seen as portraits of the neurogenomic state governing aggressiveness, with different phenotypes corresponding to distinct gene expression profiles within the central nervous system [10].

Neurotransmitters are fundamental regulators of aggressiveness. In *Portunus trituberculatus*, the interaction between serotonin (5-HT) and dopamine (DA) has been shown to mediate aggressiveness [11]. The winner and loser effect, where previous wins increase the probability of winning the next encounter while defeats decrease it, is modulated by specific neural pathways. For example, injecting 5-HT1 receptor antagonists blocks the winner effect, while the long-term memory of the loser effect relies on the cAMP-PKA (protein kinase A) signaling pathway, which regulates aggressive behavior [12,13,14]. The function and subunit composition of N-methyl-D-aspartate receptors (NMDARs) are significantly influenced by monoamine neurotransmitters [15]. These receptors orchestrate aggressive and defensive responses through calcium ion-activated calmodulin-dependent kinase II (CaMKII) [15,16,17]. CaMKII serves multiple functions: it activates tryptophan hydroxylase (the rate-limiting enzyme in 5-HT synthesis) [18,19], promotes presynaptic neurotransmitter release [20], and regulates aggressiveness [21]. Studies in mice have demonstrated that upregulation of α-CaMKII expression in the brain increases aggressive attacks in mice [22], while its absence leads to elevated defensive aggression [23]. Research on the relationship between NMDAR and aggressiveness has primarily focused on model organisms including mice [17,24,25], fruit flies [26,27,28], honeybees [7,29], and zebrafish [8,30]. In contrast, studies examining NMDAR in crustaceans remain limited (but see Hepp et al. [31,32]), particularly regarding its role in aggressiveness.

The central nervous system in crustaceans is simpler compared to that of vertebrates, consisting of fused ganglia, including the brain, suboesophageal ganglion, and thoracic ganglia [33,34]. This simplified structure serves as a classical model for understanding neural function [35,36]. The brain integrates multiple sensory inputs, such as visual information, to perceive external threats and make decisions related to aggression [37,38]. The suboesophageal ganglion acts as a relay center, connecting the brain with the thoracic ganglia [39,40] and facilitating signal transmission between threat assessment and motor responses [41,42,43]. This coordination between the thoracic ganglia and the brain ensures rapid, synchronized, and robust behavioral responses. The thoracic ganglion receives signals from the brain through these neural pathways and executes aggressive behavior by controlling appendage movements, including pereopods and chelicerae [44]. Given its essential role in behavioral execution and its strategic position within the neural network connecting the brain and suboesophageal ganglion, we selected the thoracic ganglion as our target for investigating the neural mechanisms underlying aggressiveness.

In crabs, the thoracic ganglia located in the cephalothoracic region, extend nerves to appendages and connect with the brain to coordinate movement and local reflexes [45]. Using our developed assessment model for *P. trituberculatus* [46], we can reliably identify distinct aggressiveness phenotypes. However, the neural regulatory mechanisms underlying these phenotypes remain unclear. To address this, we conducted transcriptomic and metabolomic analyses of the thoracic ganglia, identifying candidate genes and key metabolites related to aggressiveness regulation. Subsequent in vivo injection experiments were conducted to verify the relationship between aggressiveness and the NMDAR-CaMKII signaling pathway, aiming to lay the foundation for a deeper understanding of the neural mechanisms regulating aggressiveness in *P. trituberculatus*.

## 2. Results

### 2.1. Aggressive Behavior

**Figure 1:**
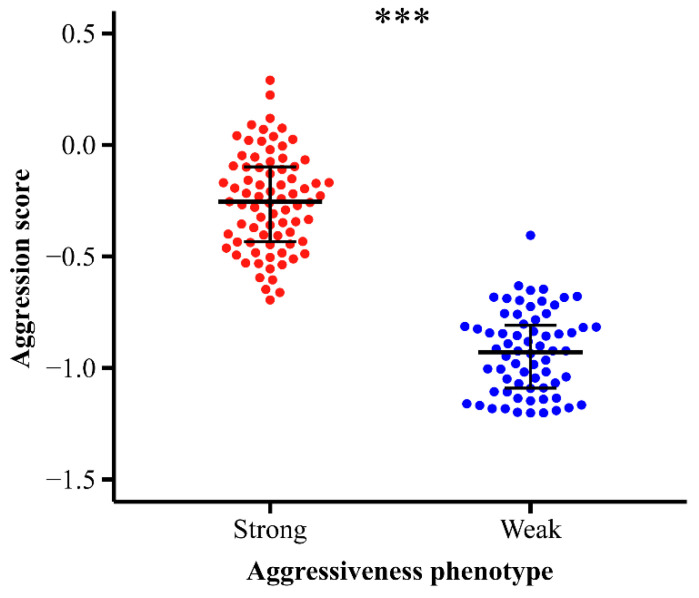
Differences in aggression scores between strong and weak aggressiveness phenotypes in swimming crabs *Portunus trituberculatus*. The horizontal coordinate represents the aggressiveness phenotype. Data were mean ± SD. The asterisk “***” indicates a very significant difference (*p* < 0.001).

**Figure 2:**
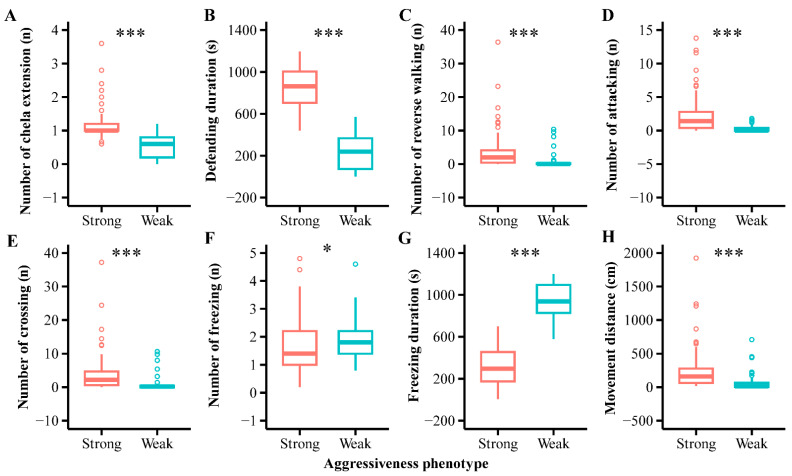
Differences in aggressive behavior between strong and weak aggressiveness of swimming crabs *Portunus trituberculatus*. (**A**) Number of chela extension. (**B**) Defending duration. (**C**) Number of reverse walking. (**D**) Number of attacking. (**E**) Number of crossing. (**F**) Number of freezing. (**G**) Freezing duration. (**H**) Movement distance. The asterisk “*” indicates a significant difference in aggressiveness (*p* < 0.05), and “***” indicates a very significant difference (*p* < 0.001).

Based on the previously established aggressiveness assessment model [46], *P. trituberculatus* was categorized into two aggressiveness phenotypes: strong and weak. The results indicated that crabs in the strong group exhibited significantly higher aggression scores than those in the weak group (*t*-test, t_144_ = 20.009, *p* < 0.001, Figure 1). Behavioral differences in aggressiveness were primarily characterized by a higher number (U-test, U = −8.935, *p* < 0.001, Figure 2A) and longer cumulative duration (*t*-test, t_144_ = 20.469, *p* < 0.001, Figure 2B) of chela extension, a higher number of attacks on the mirror image (U-test, U = −8.252, *p* < 0.001, Figure 2D), increased approaches (U-test, U = −7.407, *p* < 0.001, Figure 2E) and retreats from the mirror (U-test, U = −7.139, *p* < 0.001, Figure 2C), and a greater total movement distance (U-test, U = −7.365, *p* < 0.001, Figure 2H). Conversely, the number (U-test, U = 2.419, *p* < 0.001, Figure 2F) and cumulative duration of freezing were shorter (*t*-test, t_144_ = −21.363, *p* < 0.001, Figure 1).

### 2.2. Transcriptomic Analysis

Transcriptomic sequencing of six samples from strong and weak groups generated 39.43 Gb of clean data in total, with each sample producing over 6.07 Gb. The Q30 base percentage exceeded 95.89% (Table S2). The GCF_017591435.1 reference genome was used. Clean reads from each sample were aligned to the reference genome, with alignment rates ranging from 90.91% to 97.77%. In this study, nine DEGs were selected for qRT-PCR validation. The results demonstrated that all nine genes exhibited similar expression patterns, confirming the reliability of the transcriptomic data (Figure S1). A total of 3850 differentially expressed genes (DEGs) were identified between the strong and weak groups of *P. trituberculatus*, with 2066 genes significantly upregulated and 1784 genes significantly downregulated (*p* < 0.05, Figure S2).

GO annotation analysis revealed that the DEGs were mainly involved in cellular and metabolic processes, with the primary cellular components being the cell membrane and organelles. The molecular functions were primarily related to catalysis and binding (Figure S2). GO enrichment analysis showed that pathways were mainly concentrated on electron transport and respiratory chain-related processes, including the electron transport chain, respiratory electron transport chain, and respirasome (Figure S3). KEGG pathway enrichment analysis identified 27 significantly enriched pathways (*p* < 0.05, Table S4). The top 20 pathways were mainly associated with energy metabolism, including oxidative phosphorylation, PI3K-Akt signaling, AMPK signaling, and FoxO signaling pathways, as well as pathways of neurodegeneration (Figure S2). In the strong group, most DEGs associated with cellular energy metabolism pathways were significantly upregulated, whereas most DEGs associated with pathways of neurodegeneration were significantly downregulated (*p* < 0.05, Table S4).

**Figure 3:**
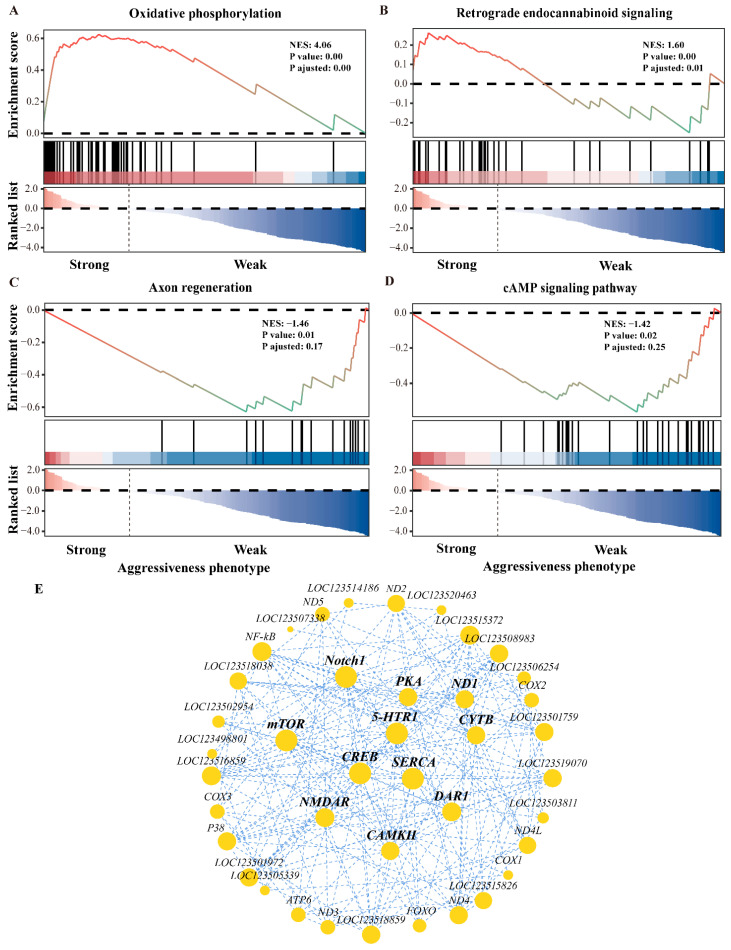
Transcriptomic analysis results of target gene sets. NES: normalized enrichment score. GS and GW represent strong and weak aggressiveness in the thoracic ganglion, respectively. (**A**–**D**) Results of GSEA analysis. The target gene sets were differentially expressed genes significantly enriched in energy metabolism and neurodegenerative diseases. The upper curves show cumulative Enrichment Score (ES) trends, with the peak indicating the ES value of the gene set. The middle vertical lines indicate the positions of genes from the predefined set within the ranked gene list. Each gene in the predefined set is marked by a black line indicating its position in the ranked gene list. The heatmap illustrates gene expression, with red indicating genes highly expressed in the GS group and blue indicating genes highly expressed in the GW group. (**E**) Correlation of gene expression within the target gene set. Nodes represent genes, and lines indicate significant correlations between them. The size of each node reflects its degree, which represents the number of correlations between that gene and other genes in the network. Larger nodes indicate genes with more correlations.

Using the criteria of |NES| > 1 (normalized enrichment score) and *p adjust* < 0.25, gene set enrichment analysis (GSEA) of DEGs enriched in energy metabolism and neurodegeneration identified key signaling pathways, including oxidative phosphorylation, retrograde endocannabinoid, axon regeneration, and cAMP (Figure 3A–D). Correlation analysis of gene expression levels, which contributed most to the GSEA enrichment scores, identified key genes associated with aggressiveness phenotypes, including *N-methyl-D-aspartate receptor 2B* (*NR2B*), *Calcium ion activation of calmodulin-dependent kinase II* (*CaMKII*), *Cyclic AMP-responsive element-binding protein* (*CREB*), *cAMP-dependent protein kinase catalytic subunit 1* (*PKA*), *Calcium-transporting ATPase sarcoplasmic/endoplasmic reticulum type (SERCA)*, *5-HT receptor1* (*5-HTR1*), *DA receptor1* (*DAR1*), *Cytochrome b* (*CYTB*), *NADH-ubiquinone oxidoreductase chain 1* (*ND1*), *Mammalian target of rapamycin* (*mTOR*), and *Neurogenic locus Notch protein* (*Notch1*) (Figure 3E). In summary, differences in the aggressiveness phenotypes of *P. trituberculatus* may be closely linked to neuronal function and structure, oxidative phosphorylation, protein synthesis and metabolism, and intercellular signaling.

### 2.3. Metabolomics Analysis

**Figure 4:**
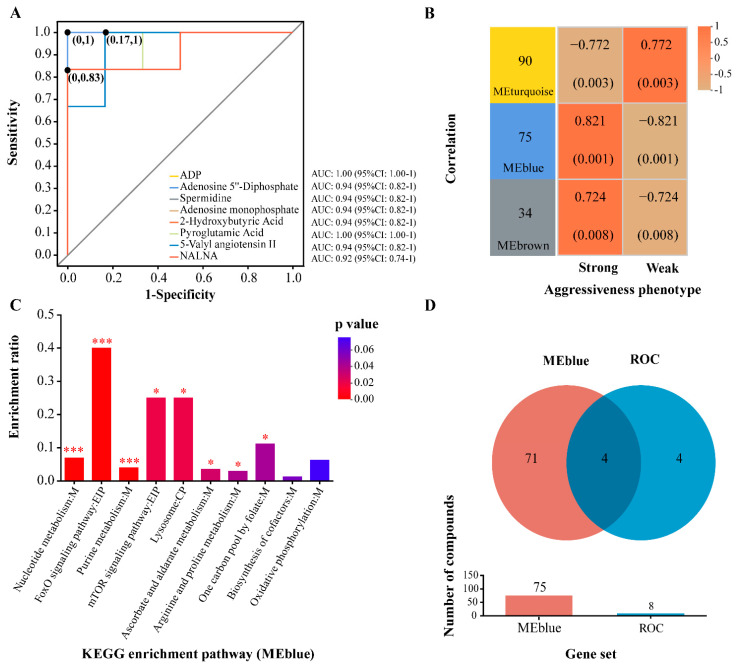
Receiver operating characteristic (ROC) and weighted gene co-expression network analysis (WGCNA) of differential metabolite sets in strong and weak aggressiveness phenotypes. (**A**) ROC analysis results. The ROC is commonly used to evaluate classification model performance by measuring sensitivity and specificity across thresholds. The *y*-axis represents the true positive rate (sensitivity), while the *x*-axis represents the false positive rate (1-specificity). The area under the curve (AUC) represents the area under the ROC curve, with an AUC above 0.9 signifying high accuracy. The CI represents the 95% confidence interval of the AUC, calculated via non-parametric resampling methods. Points on the curve mark the optimal threshold for distinguishing between two classes. (**B**) WGCNA module-phenotype correlations. The *x*-axis represents different aggressiveness phenotypes, while the *y*-axis represents different modules. Numbers on the left show the number of metabolites per module. The right side displays correlation coefficients and significance (*p*-value) for each module-phenotype pair. Larger absolute values indicate stronger correlations. Red denotes positive correlations and blue denotes negative correlations. (**C**) KEGG pathway enrichment analysis for MEblue module metabolites. The *x*-axis lists pathway names, while the *y*-axis represents the enrichment ratio, indicating the proportion of metabolites enriched in the pathway compared to the total annotated metabolites. The color gradient of the bars reflects enrichment significance, with darker colors indicating higher significance. Pathways with *p* < 0.001 are marked as “***”, and *p* < 0.05 as “*”. (**D**) Venn diagram of differential metabolites in MEblue and ROC gene sets. It illustrates differential metabolites identified by ROC analysis and WGCNA. Different colors represent distinct groups, with overlapping areas indicating shared metabolites between ROC and WGCNA groups. Non-overlapping areas represent unique metabolites in each group. The bar chart displays the number of metabolites in each set.

LC-MS analysis identified 1914 metabolites in positive ion mode and 1257 metabolites in negative ion mode. OPLS-DA discriminant analysis showed model evaluation parameters of R^2^X = 0.393, R^2^Y = 1, and Q^2^ = 0.816, indicating that the model is stable, reliable, and has strong fitting and predictive capabilities. The OPLS-DA model effectively differentiated the two groups (Figure S4). Using VIP > 1 and *p* < 0.05 as criteria, 199 differential metabolites (DMs) were identified between the strong and weak groups, with 109 DMs significantly upregulated and 90 DMs downregulated (Table S5). The DMs primarily included nucleotides and derivatives like ADP, AMP, and IMP; amines and biogenic amines such as spermidine, MDPA, and N-Methyl-14-O-demethylepiporphyroxine; and hormones and peptides, including 5-Valyl Angiotensin II, methyltestosterone, and L-thyronine. Receiver operating characteristic (ROC) analysis of these key DMs showed that eight metabolites had an area under the curve (AUC) > 0.9, including ADP, spermidine, and AMP (Figure 4A).

KEGG pathway enrichment analysis identified 34 enriched metabolic pathways, 10 of which were significantly enriched. These pathways mainly included nucleotide metabolism (nucleotide, pyrimidine, purine metabolism) and signal transduction pathways (phosphatidylinositol, FoxO, mTOR signaling) (Figure S4). In the strong group, 17 metabolic pathways, including oxidative phosphorylation, FoxO signaling, and lysosome pathways, were upregulated, whereas only glycerophospholipid metabolism and phosphatidylinositol signaling were significantly downregulated (Figure S4). WGCNA of the DMs identified three metabolic modules. The MEturquoise module was significantly positively correlated with the weak-aggressiveness phenotype (R = 0.772, *p* = 0.003), while the MEblue (R = 0.821, *p* = 0.001) and MEbrown (R = 0.724, *p* = 0.008) modules were positively correlated with the strong-aggressiveness phenotype. The MEblue module showed the strongest correlation with the aggressiveness phenotype (Figure 4B). KEGG pathway enrichment analysis of the MEblue module’s metabolic set revealed significant enrichment in eight pathways, including FoxO signaling, mTOR signaling, nucleotide metabolism, purine metabolism, and lysosome metabolism, all of which were significantly enriched in both KEGG analyses (Figure 4C). Venn analysis between the DMs identified in the ROC analysis and those in the MEblue module revealed three key metabolites associated with aggressiveness phenotype: ADP, spermidine, and 2-hydroxybutyric acid, all significantly upregulated in the strong group (*p* < 0.05, Figure 4D). These metabolites primarily function in cellular energy metabolism, stress response, and cell protection.

### 2.4. Integrated Analysis of Transcriptomics and Metabolomics

Two-way orthogonal partial least square (O2PLS) analysis resulted in R^2^X = 0.694 and R^2^Y = 0.663, indicating the model explains 69.4% of the variance in transcriptomics data and 66.3% in metabolomics data, demonstrating a good fit (Figure S5). A clear separation of samples with strong and weak aggressiveness along the first principal component indicates significant differences in transcriptomic and metabolomic data. The top 20 KEGG pathways enriched for differentially expressed genes and metabolites between strong- and weak-aggressiveness *P. trituberculatus* primarily involved metabolism (Figure S5), including oxidative phosphorylation, pyrimidine metabolism, and purine metabolism. Additionally, some pathways were related to environmental information processing and cellular regulation, including lysosome, FoxO signaling, mTOR signaling, neuroactive ligand-receptor interaction, and phosphatidylinositol signaling, with significant co-enrichment in FoxO and mTOR signaling pathways (*p* < 0.05, Figure S5). In these enriched signaling pathways, key metabolites ADP and AMP showed significantly higher expression levels in the strong-aggressiveness crabs (Table S5).

### 2.5. Regulation of Aggressiveness by the NMDAR-CaMKII Pathway

**Figure 5:**
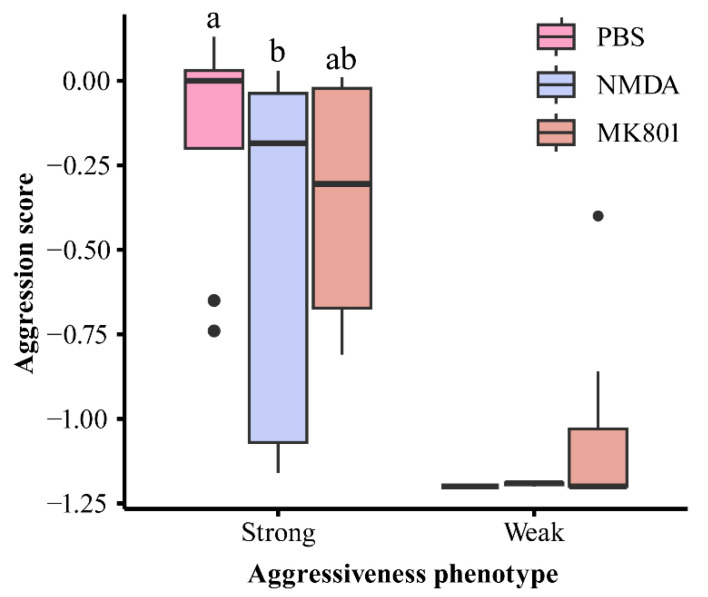
Variance in aggression scores of swimming crabs *Portunus trituberculatus* with different aggressiveness after experimental treatment. Different lowercase letters indicate significant differences in the same aggressiveness phenotype of *P. trituberculatus* under various experimental treatments (*p* < 0.05). Black dots represent outliers.

As shown in Figure 5, aggressiveness trends differed among *P. trituberculatus* phenotypes following NMDAR activator (NMDA) and inhibitor (MK801) injections. Compared to the PBS group, NMDA injection significantly reduced aggressiveness in the strong group (H-test, H = 9.417, *p* < 0.05) and decreased it in the weak group. Similarly, MK801 injection decreased aggressiveness in the strong group while increasing it in the weak group.

**Figure 6:**
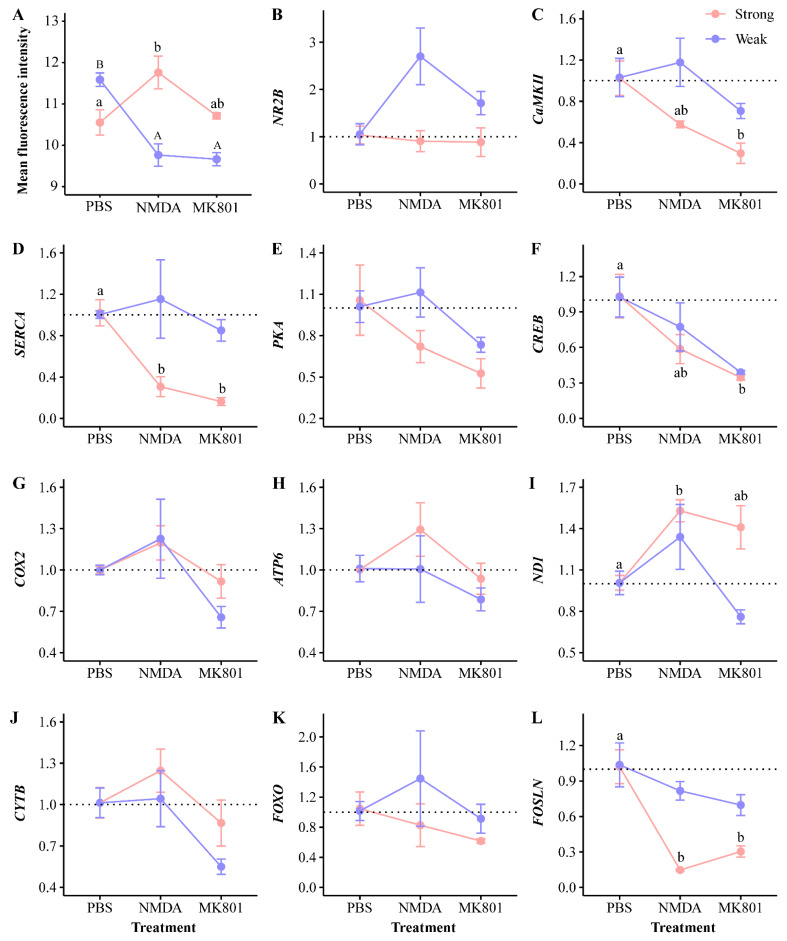
Results of NMDAR-CaMKII pathway validation. Differences in mean fluorescence intensity and relative expression levels of key genes were observed across aggressiveness phenotypes of swimming crabs *Portunus trituberculatus* after PBS, NMDA (NMDAR agonist), and MK801 (NMDAR antagonist) injections. Different uppercase or lowercase letters indicate significant differences within the same aggressiveness phenotype of *P. trituberculatus* across experimental treatments (*p* < 0.05). (**A**) Mean fluorescence intensity differences of NMDAR protein. (**B**–**L**) Mean fold changes in relative expression levels of key genes. The *x*-axis represents experimental treatments, and the *y*-axis shows mean fold changes in gene expression. The dashed line indicates a fold change of 1; values above 1 indicate gene upregulation, while values below 1 indicate gene downregulation.

**Figure 7:**
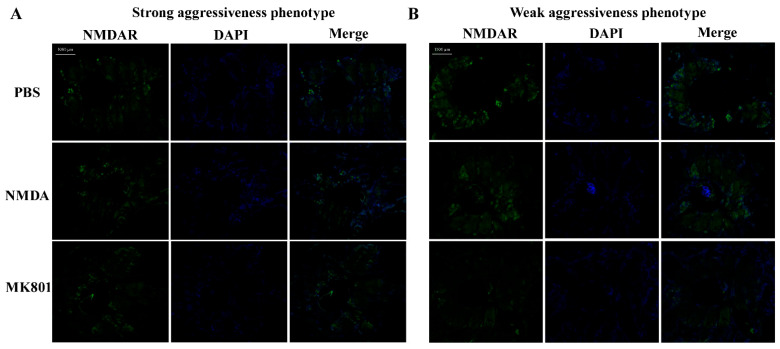
Fluorescent single plots of strong (**A**) and weak (**B**) aggressiveness phenotypes in the thoracic ganglia. NMDAR fluorescence intensity (488 channel) is shown in green. The DAPI channel is shown in blue.

After NMDA injection, the *NR2B* gene was upregulated in the weak group compared to the PBS group, while NMDAR protein fluorescence intensity significantly decreased (one-way ANOVA, F_2,6_ = 23.787, *p* < 0.01, Figure 6 and Figure 7). Aggressiveness-related genes like *CaMKII*, *SERCA*, *PKA*, *COX2*, *ND1*, *CYTB*, and *FOXO*, were upregulated, whereas *CREB* and *FOSLN* were downregulated. In the strong group, *NR2B* was downregulated, and NMDAR protein fluorescence intensity significantly increased (one-way ANOVA, F_2,6_ = 5.475, *p* < 0.05, Figure 6 and Figure 7). *CaMKII*, *SERCA*, *PKA*, *CREB*, *FOXO*, and *FOSLN* were downregulated, whereas oxidative phosphorylation-related genes like *COX2*, *ATP6*, *ND1*, and *CYTB* were upregulated.

Following MK801 injection, *NR2B* was upregulated in the weak group compared to the PBS group, with a significant decrease in NMDAR protein fluorescence intensity (one-way ANOVA, F_2,6_ = 23.787, *p* < 0.01, Figure 6 and Figure 7). All 11 key aggressiveness-related genes were downregulated. In the strong group, *NR2B* was downregulated, and NMDAR protein fluorescence intensity increased. *CaMKII* (one-way ANOVA, F_2,6_ = 10.449, *p* < 0.05), *SERCA* (one-way ANOVA, F_2,6_ = 23.637, *p* < 0.01), *CREB* (one-way ANOVA, F_2,6_ = 7.410, *p* < 0.05), and *FOSLN* (one-way ANOVA, F_2,6_ = 28.442, *p* < 0.01) genes were significantly downregulated (Figure 6). *PKA*, *COX2*, *ATP6*, and *CYTB* were also downregulated, while *ND1* was significantly upregulated (one-way ANOVA, F_2,6_ = 6.656, *p* < 0.05).

## 3. Discussion

This study began by assessing behavioral differences between strongly and weakly aggressive phenotypes in *P. trituberculatus* swimming crabs. Transcriptomic and metabolomic analyses of the thoracic ganglia revealed that genes associated with aggressiveness were primarily enriched in pathways related to energy metabolism and neural behavior regulation. Further screening identified eleven candidate genes (e.g., *NR2B*, *CaMKII*, and *CREB*) and three key metabolites (ADP, spermidine, and 2-hydroxybutyric acid) associated with aggressiveness. Among these, ADP was identified as a biomarker in the thoracic ganglia for determining aggressiveness levels in *P. trituberculatus*. In vivo injection experiments demonstrated that NMDAR is a critical receptor in the regulation of aggressiveness. These findings suggest that the NMDAR-CaMKII pathway plays a pivotal role in controlling aggressiveness in *P. trituberculatus* by modulating neural energy states. This regulation occurs through cascades such as oxidative phosphorylation, cAMP, FoxO, and axon regeneration signaling pathways, which ultimately affect aggressive behavior.

### 3.1. Aggressiveness Phenotypes and NMDAR-CaMKII Pathway

The NMDAR-CaMKII signaling pathway is closely related to the regulation of neural behavior. Specifically, the NMDAR 2B subunit (NR2B) is a crucial functional component of NMDAR, triggering Ca^2+^ influx and leading to the phosphorylation of CaMKII and CREB [47,48]. Previous rodent studies have demonstrated that overexpression of the *α-CaMKII* gene enhances aggressiveness [22,23]. CaMKII-mediated aggression is linked to reduced extracellular glutamate clearance and increased NMDAR excitability, emphasizing the importance of NMDAR in the regulation of aggressiveness [49]. These studies suggest that NMDAR plays a critical role in mediating CaMKII regulation of aggressiveness, consistent with our findings. In this study, significant differences were observed in the relative expression levels of *NR2B*, *CaMKII*, and *CREB* genes (Figure 3), as well as in the fluorescence intensity of NMDAR protein, between the different aggressiveness phenotypes. NMDAR activation led to significant decreases in the expression levels of *CaMKII* and *CREB* genes, resulting in decreased aggressiveness in *P. trituberculatus* (Figure 5 and Figure 6).

The modulation of aggressive behavior in crustaceans involves coordinated interactions between multiple regions of the nervous system, particularly the brain and ganglia [50]. The brain processes sensory information and emotional responses to guide aggression-related decision-making, subsequently modulating motor functions through neural circuits connected to the thoracic ganglia [51,52]. The thoracic ganglion coordinates appendage movements including walking legs and chelipeds to execute aggressive behaviors [44]. While the role of the thoracic ganglion in aggression is distinct from that of the brain, both components are essential and complementary in the neural regulation of behavior. The thoracic ganglion is regulated by neurotransmitters such as dopamine (DA) and serotonin (5-HT) while maintaining tight functional links with aggression-related circuits in the brain [53]. When a crab perceives a threat, the dopamine system in the brain activates the aggression circuit, prompting the thoracic ganglion to direct aggressive movements of chelipeds and walking legs [13]. In addition, the thoracic ganglion serves as a key intermediary, transmitting signals between the brain and peripheral ganglia through neurotransmitter regulation. The NMDAR-CaMKII pathway, critical for brain development and synaptic plasticity [54,55,56], has been linked to aggressiveness through studies of genes involved in neurogenesis and synaptogenesis [57,58,59,60]. Based on these findings, we propose that this pathway is also involved in regulating aggressiveness in the thoracic ganglia of *P. trituberculatus*, with NMDAR serving as a key target protein.

NMDAR-mediated excitability may be linked to the regulation of neural behavior. Studies in humans have demonstrated that alterations in NMDAR can lead to neurodegenerative diseases like Alzheimer’s disease [61,62], often accompanied by mitochondrial dysfunction [63,64]. Our KEGG enrichment analysis revealed that the differentially expressed genes (DEGs) between aggressiveness phenotypes of *P. trituberculatus* were significantly enriched in pathways related to neurodegenerative diseases (Figure S2). In the weak group, the relatively upregulated NMDAR-CaMKII pathway triggered a series of cellular responses and metabolic changes in the nervous system. Specifically, key regulatory genes involved in mitochondrial oxidative phosphorylation, such as *ND1*, *COX2*, *CYTB*, and *ATP6*, were significantly downregulated, along with a notable reduction in the levels of key metabolites ADP and AMP (Tables S3 and S5). This suggests that in the nervous system of weak-aggressiveness crabs, the downregulation of energy metabolism-related genes and metabolites induced by the NMDAR-CaMKII pathway may be linked to the upregulation of neurodegenerative disease pathways. One possible explanation is that the relationship between aggressiveness and disease-inducing genes represents a trade-off between two energy-demanding phenotypes. Strong-aggressiveness individuals invest more in neural energy regulation, whereas weak-aggressiveness individuals allocate more resources to immune function [65,66]. Previous studies on honeybees (*Apis mellifera*) found that weakly aggressive individuals are more sensitive to localized pesticide treatment [67] and exhibit lower colony activity [68]. However, pathogens were not significantly enriched in samples from weakly aggressive individuals, suggesting that the molecular differences regulating aggressiveness were not due to pathogen infection [66]. Metabolomics results also indicated that neurotransmitter levels, including serotonin, norepinephrine, and glutamate, in the thoracic ganglia did not significantly differ between the various aggressiveness phenotypes of *P. trituberculatus* (Table S5). This indicates that although the pathway is upregulated, NMDAR is not excessively activated and remains within the normal range of expression differences among phenotypes is naturally aggressive. When exposed to prolonged stress, weakly aggressive individuals exhibit reduced activity, and their physiological state may incline them to invest in health maintenance rather than aggressiveness, thereby reducing cellular energy demands.

### 3.2. Aggressiveness Phenotypes and Neural Energy State

The neural energy state, defined by the activation of metabolic pathways such as glycolysis and oxidative phosphorylation, can predict the outcomes of social interactions [69]. The NMDAR-CaMKII pathway appears to regulate emotional states, such as anxiety, through its modulation of neurotransmitter release and neuronal activity [70]. Behavioral regulation at the neural level often involves neuroactive compounds, including neurotransmitters, neuromodulators, and neurohormones, which can enhance or attenuate signaling. Individual behavioral differences arise from changes in endogenous neurotransmitter production or variations in receptor quantity or activity [71,72,73]. In honeybees (*Apis mellifera*), serotonin and dopamine regulate neural energy states in brain regions linked to aggressiveness. Higher mitochondrial density characterizes these regions, which are central to aggressive responses [69]. Similarly, serotonin and dopamine enhance aggressiveness in *P. trituberculatus* in a dose-dependent manner [11]. In this experiment, significant differences in the relative expression of *5-HTR1* and *DAR1* genes were observed between aggressiveness phenotypes (Figure 3). Aggression-related genes were mainly enriched in energy metabolism-related pathways, including oxidative phosphorylation, PI3K-Akt, AMPK, and FoxO signaling pathways (Figure S2). In the thoracic ganglia of highly aggressive *P. trituberculatus*, we observed elevated expression of genes related to mitochondrial oxidative phosphorylation, along with increased levels of ADP and AMP (Tables S3 and S5). This indicates that more aggressive individuals have greater neuronal energy demands, suggesting a positive correlation between the aggression level and neural energy state in *P. trituberculatus*. To meet these elevated energy requirements, organisms upregulate electron transport chain activity and oxidative phosphorylation in neuronal mitochondria.

The metabolic differences between aggressiveness phenotypes may stem from multiple underlying factors. In the thoracic ganglia of *P. trituberculatus*, highly aggressive individuals show downregulated FoxO pathway activity and upregulated oxidative phosphorylation, indicating possible metabolic reprogramming and a shift between metabolic states. The significant upregulation of *G6PC1* (glucose-6-phosphatase catalytic subunit 1) and downregulation of *PFKFB1* (fructose-2,6-bisphosphatase 1), coupled with higher ADP and AMP levels, increased NAD^+^ levels, and lower lactate levels (Tables S3 and S5), suggest a shift in the neural energy state of highly aggressive crabs from glycolysis to gluconeogenesis and oxidative phosphorylation. Previous studies have linked changes in energy metabolism to neurodegenerative disease pathways [74]. This supports our conclusion that the downregulation of energy metabolism-related genes and metabolites, induced by the NMDAR-CaMKII pathway in the thoracic ganglia, may correlate with the upregulation of neurodegenerative disease pathways.

Increased neuronal energy demand is linked to enhanced lysosomal autophagy. The mammalian target of rapamycin (mTOR) and AMP-activated protein kinase (AMPK) are crucial regulators of autophagy initiation and autophagosome formation [75]. This study found that differentially expressed genes and metabolites between aggressiveness levels in *P. trituberculatus* were significantly co-enriched in lysosomal and mTOR signaling pathways (Figure S5). Notably, *AMP* and *AMPK* genes were significantly upregulated, while the *mTOR* gene was downregulated (Table S3). AMPK functions as the cell’s energy sensor, detecting the ATP/AMP ratio, while the mechanistic Target of Rapamycin Complex 1 (mTORC1) regulates metabolism by sensing cellular energy and nutrient levels, with mTOR as a key component. Reduced mTORC1 activity enhances autophagy. In highly aggressive individuals, increased neuronal energy demand leads to greater AMPK activation, which promotes autophagy and inhibits mTORC1, thereby increasing energy production and reducing consumption. Furthermore, AMPK also promotes mitochondrial biogenesis and function, enhancing the efficiency of oxidative phosphorylation [76]. Mitochondria can then use autophagic products for oxidative phosphorylation to generate ATP [77]. These gene expression patterns represent an adaptive response to environmental and behavioral stressors, allowing the nervous system to meet the high energy demands during aggressive behavior.

### 3.3. Limitations and Perspectives

Aggressive behavior in crustaceans arises from the complex interplay of multiple neural structures and neurotransmitter systems. In *P. trituberculatus*, the thoracic ganglion, responsible for coordinating motor and physiological control during aggressive behaviors, precisely orchestrating the movements of pereopods and chelipeds in predation, attack, and escape. While this ganglion is vital for executing aggressive actions, the role of the brain in processing sensory input and decision-making is equally important. A key limitation of our study is the absence of brain tissue analysis, which necessitates future integrated studies to fully elucidate the neuromodulatory network underlying aggressiveness.

## 4. Methods

### 4.1. Animal Collection and Maintenance

Male swimming crabs, *P. trituberculatus* (carapace width 91.32 ± 4.27 mm), were purchased from an aquaculture farm in Jiaonan, Shandong Province. During transport, the water temperature was kept at approximately 24 °C, with dissolved oxygen levels maintained above 7 mg/L to minimize environmental stress. The crabs were individually housed in aquariums (45 × 30 × 30 cm, 40.95 L) and acclimated for two weeks at the laboratory. The water was maintained at 24 ± 1 °C, salinity at 30, dissolved oxygen above 7 mg/L, and a photoperiod of 12L:12D was used. Each day at 19:00, the crabs were fed sufficient live Manila clams (*Ruditapes philippinarum*). Residual food and feces were removed at 07:00 the next day, and one-third of the seawater in each aquarium was replaced daily. The crabs were fasted for 24 h before the formal experiments, and individuals with intact appendages in the intermolt stage were randomly selected for subsequent experiments.

### 4.2. Experimental Design

This experiment was conducted in two parts. In the first part, the crabs with different phenotypes were selected for analyzing behavioral differences and then conducting transcriptomic and metabolomic analyses of the thoracic ganglia. The second part involves validating the NMDAR-CaMKII pathway. The sequencing samples for the first part are a subset of a study conducted by our research group in the summer of 2022, where we proposed a method to assess aggressiveness in *P. trituberculatus* [46]. We quantified the indicators of aggressiveness, including chela extension, reverse walking, attacking, crossing, freezing, defending, and movement distance. For definitions of these indicators, see Liang et al., 2024 [46]. Based on this, 146 *P. trituberculatus* crabs, comprising strong (n = 79) and weak (n = 67) phenotypes, were selected for aggressive behavior analysis and omics analysis (behavior measurement methods in Liang et al. [46]). For omics analysis, after measuring aggressiveness, the crabs were anesthetized by freezing for 5 min. They were then quickly dissected on ice, and their thoracic ganglia were removed. The ganglia were placed in 2 mL cryogenic vials, flash-frozen in liquid nitrogen, and stored at −80 °C for later analysis.

#### 4.2.1. Transcriptome Sequencing

Each group, representing strong and weak aggressiveness, had three biological replicates, with two swimming crabs per replicate. Total RNA was extracted using the Trizol method. RNA concentration and purity were assessed using a Nanodrop2000 (Thermo Fisher Scientific, Qingdao, China). RNA integrity was verified by agarose gel electrophoresis. The RNA Integrity Number (RIN) was determined using an Agilent 2100 (Agilent Technologies, Qingdao, China). Following the extraction of 1 µg of total RNA, magnetic beads with Oligo (dT) were used to bind the poly-A tail at the 3′ end of mRNA. The mRNA was purified, followed by the addition of fragmentation buffer to randomly break it into ~300 bp fragments. The purified mRNA served as a template for reverse transcription of the first cDNA strand, followed by synthesis of the second strand. An end repair mix was added to create blunt ends, followed by the addition of an “A” base to the 3′ ends. The adapter-ligated products were purified and size-selected, then amplified by PCR, resulting in the final purified library. The constructed library was sequenced on DNBSEQ-T7 platform(PE150, BGI Genomics, Shenzhen, China) using the DNB-SEQ-T7RS Reagent Kit (FCL PE150, BGI Genomics, Shenzhen, China) version 3.0. Statistical methods assessed the quality of each cycle of sequencing reads. SeqPrep (v1.3) and Sickle software (v1.33) filtered the raw sequencing data to obtain high-quality clean data. HiSat2 (v2.2.1) and TopHat2 (v2.1.1) software were used to align the quality-controlled clean data to the reference genome, followed by a quality assessment of the alignment results. RSEM software (v1.3.3) quantified gene and transcript expression levels, calculated in fragments per kilobase of transcript per million mapped reads (FPKMs). The criteria for selecting significantly differentially expressed genes (DEGs) were: FDR < 0.05 and |log2FC| ≥ 2. All sequencing work was conducted at Shanghai Majorbio Bio-Pharm Technology Co., Ltd., Shanghai, China.

#### 4.2.2. Metabolome Assay

Both the strong and weak aggressiveness groups had six biological replicates, each containing two swimming crabs. We weighed exactly 50 mg of thoracic ganglia tissue and placed it in 2 mL centrifuge tubes. Metabolites were extracted with 400 µL of an extraction solution consisting of methanol and water at a 4:1 ratio, containing 0.02 mg/mL of the internal standard L-2-chlorophenylalanine. The sample solution was ground at −10 °C using a cryogenic grinder at 50 Hz for 6 min, followed by ultrasonic extraction at 5 °C and 40 kHz for 30 min. The processed samples were left to stand at −20 °C for 30 min, centrifuged at 13,000× *g* and 4 °C for 15 min, and the supernatant was collected for further analysis. Chromatographic analysis was conducted using an HSS T3 column (100 mm × 2.1 mm i.d., 1.8 µm). Mass spectrometry analysis was performed in both positive and negative ion scanning modes, covering a mass scan range of 70–1050 *m*/*z*. The sheath gas flow rate was set at 50 psi, the auxiliary gas flow rate at 13 psi, and the auxiliary gas heating temperature at 425 °C. The ion spray voltage was set to ±3.5 kV for both positive and negative modes. Data acquisition was conducted in the data-dependent acquisition mode.

The raw LC-MS data were processed using progenesis QI software (v2.3, Waters Corporation, Milford, CT, USA), encompassing baseline filtering, peak recognition, integration, retention time correction, and peak alignment. Metabolite information was acquired by matching with public metabolic databases such as HMDB, Metlin, and Majorbio’s proprietary database. Orthogonal partial least squares discriminant analysis (OPLS-DA) was conducted using the “ropls” package in R, with model stability assessed via 7-fold cross-validation. Differential metabolites were identified using VIP values from the OPLS-DA model and *p*-values from the Student’s *t*-test, with thresholds of VIP > 1 and *p* < 0.05. Differential metabolites (DEs) were structurally identified through databases like KEGG, and their roles in metabolic pathways were determined. Pathway enrichment analysis was carried out using the “scipy. stats” package in Python, with Fisher’s exact test employed to identify the biological pathways most relevant to the experimental treatment. Metabolites were linked to phenotype data via WGCNA to identify key modules.

#### 4.2.3. NMDAR-CaMKII Pathway Validation Experiments

After the temporary rearing period, 60 swimming crabs in the intermolt stage, all with intact appendages, were randomly selected. Their initial aggressiveness was measured through mirror experiments following a 24 h fasting period. Each crab underwent the mirror experiment three times. The experiment was conducted in a dark environment. The interior walls of the observation tank were white and opaque, ensuring no interference between different recording systems. During the experiment, water temperature and salinity were maintained consistent with those during the temporary rearing period. The recording room was equipped with eight behavior collection devices, enabling simultaneous recording of eight groups. All recordings were conducted during the day without any artificial interference in the recording room.

After a recovery period of at least 24 h, the drug injections were administered. The experimental groups received injections of an NMDA activator (NMDA, HY-17551) and an NMDA receptor inhibitor (MK801, HY-15084), while the control group was injected with PBS. Each treatment group consisted of 20 crabs, comprising 10 strong and 10 weak individuals. A pointed micro-injector (250 µL) was used to inject 100 µL at the base of the swimming leg at a concentration of 0.1 mM and a depth of 4 mm, ensuring the solution was delivered into the pericardial cavity and fully released. Aggressiveness was measured and assessed 15 min post-injection. Following the behavioral assessment, the crabs were immediately anesthetized by freezing, quickly dissected on ice, and their thoracic ganglia were placed in 2 mL cryogenic vials. One portion was flash-frozen in liquid nitrogen and stored at −80 °C for future analysis, while the other portion was fixed in 4% paraformaldehyde for 24 h, dehydrated in graded ethanol, cleared in xylene, embedded in paraffin, and sectioned at a thickness of approximately 5 µm. The tissue sections were mounted on anti-detachment slides and baked at 37 °C overnight for subsequent immunofluorescence experiments.

### 4.3. Tissue Immunofluorescence Assay

The paraffin sections were initially treated with eco-friendly dewaxing solutions I, II, and III for 10 min each, then rehydrated in graded anhydrous ethanol. The sections then underwent antigen retrieval in pH 6.0 citrate buffer using a microwave: 8 min at medium power, an 8 min pause, and 7 min at medium-low power, followed by natural cooling. The slides were washed in PBS (pH 7.4), blocked with 3% BSA for 30 min, and then incubated overnight at 4 °C with a primary antibody diluted 1:50 (Sigma-Aldrich, St. Louis, MO, USA; Cat# G8913, RRID:AB_259978). The next day, after washing, the sections were incubated with a secondary antibody diluted 1:400 (ServiceBio, Wuhan, China; Cat# GB25303, RRID:AB_2910224) at room temperature for 50 min in the dark. DAPI staining was used for nuclear counterstaining, and incubated at room temperature for 10 min in the dark. To quench autofluorescence, the sections were treated with quenching reagent B for 5 min, followed by a 10 min rinse in running water. Finally, the slides were mounted with an anti-fade medium and observed under a fluorescence microscope using DAPI (blue) and 488 (green) channels for imaging.

### 4.4. Quantitative Real-Time PCR Analysis

Total RNA was extracted from *P. trituberculatus* tissues using the Trizol method. RNA integrity was assessed using 1% agarose gel electrophoresis. RNA concentration and purity were measured using a Nanodrop 2000 spectrophotometer, ensuring an A260/A280 ratio between 1.8 and 2.2. cDNA was synthesized via reverse transcription using the HiScript^®^ III RT SuperMix Kit (Vazyme Biotech Co., Ltd. R323, Nanjing, China). Real-time quantitative PCR was conducted with the Taq Pro Universal SYBR qPCR Master Mix Kit (Vazyme Biotech Co., Ltd. Q712, Nanjing, China). Primers were designed using Primer5 software (Table S1), and their amplification efficiency was validated to be between 1.8 and 2.2. *β*-actin served as the reference gene. Each sample was tested in three technical replicates. Quantitative PCR results were analyzed using the 2^−ΔΔCt^ method [78]. Kits used in this experiment were provided by Vazyme Biotech Co., Ltd. (Nanjing, China), and primers were synthesized by Sangon Biotech (Shanghai, China) Co., Ltd.

### 4.5. Statistical Analysis

Statistical analyses were conducted using SPSS 26.0 and R 4.2.3. Data were first assessed for homogeneity of variance using Levene’s test and for normality using the Kolmogorov–Smirnov test. For comparison of aggressive phenotypes, independent samples *t*-tests were used for normally distributed variables (aggression scores, and durations of defending and freezing behaviors), while Mann–Whitney U-tests were employed for non-normally distributed variables (frequencies of chela extension, reverse walking, attacking crossing, and freezing, as well as movement distance). In NMDAR activator and inhibitor injection experiments, the expression of eleven aggressiveness-related genes and NMDAR protein fluorescence intensity was analyzed using analysis of variance (ANOVA), as these data followed a normal distribution. Aggression scores from the injection experiments were analyzed using the Kruskal–Wallis H-test due to non-normal distribution. A significance level of *p* < 0.05 was set for all analyses.

## 5. Conclusions

This study identified eleven candidate genes (e.g., *NR2B*, *CaMKII*, and *CREB*) and three key metabolites (e.g., ADP) associated with aggressiveness phenotypes in *P. trituberculatus*, revealing a correlation between the NMDAR-CaMKII signaling pathway and aggression regulation. Our findings suggest that in the central nervous system of *P. trituberculatus*, NMDAR acts as a key receptor whose protein expression may be inversely correlated with aggression levels. This pathway appears to influence the neuronal energy state by modulating pathways such as oxidative phosphorylation, cAMP signaling, FoxO signaling, and axon regeneration, thereby regulating aggressiveness phenotypes. Furthermore, we observed that a downregulation of neuronal energy states may be linked to the upregulation of pathways associated with neurodegeneration, suggesting a potential connection between aggression and genes involved in neurodegeneration. The relationship between aggression and health in crustaceans remains largely unexplored and warrants further investigation.

## Data Availability

All data generated or analyzed during this study are included in this published article, its Supplementary Information files and publicly available repositories. The dataset supporting the conclusions of this article is available in the National Center for Biotechnology Information repository, the accession number is PRJNA1137250.

## Supplementary Materials

The following supporting information can be downloaded at: https://www.mdpi.com/article/10.3390/ijms252312560/s1.

## References

[1] Rao Z., Cao L., Wu H., Han R. (2021). Transcriptome analyses provide insights into the aggressive behavior toward conspecific and heterospecific in *Thitarodes xiaojinensis* (Lepidoptera: Hepialidae). Insects.

[2] Dashtbali M., Long X., Henshaw J.M. (2024). The evolution of honest and dishonest signals of fighting ability. Evol. Lett..

[3] Sih A., Bell A.M., Johnson J.C., Ziemba R.E. (2004). Behavioral syndromes: An integrative overview. Q. Rev. Biol..

[4] Reale D., Reader S.M., Sol D., McDougall P.T., Dingemanse N.J. (2007). Integrating animal temperament within ecology and evolution. Biol. Rev..

[5] Briffa M., Elwood R.W. (2005). Rapid change in energy status in fighting animals: Causes and effects of strategic decisions. Anim. Behav..

[6] Arnott G., Elwood R.W. (2009). Assessment of fighting ability in animal contests. Anim. Behav..

[7] Paula G.M., da Silva Menegasso A.R., Dos-Santos-Pinto J.R.A., Malaspina O., Palma M.S. (2024). Profiling the neuroproteomics of honeybee brain: A clue for understanding the role of neuropeptides in the modulation of aggressivity. J. Proteom..

[8] Reichmann F., Pilic J., Trajanoski S., Norton W.H.J. (2022). Transcriptomic underpinnings of high and low mirror aggression zebrafish behaviours. BMC Biol..

[9] Dierick H.A., Greenspan R.J. (2006). Molecular analysis of flies selected for aggressive behavior. Nat. Genet..

[10] Kudo A., Shigenobu S., Kadota K., Nozawa M., Shibata T.F., Ishikawa Y., Matsuo T. (2017). Comparative analysis of the brain transcriptome in a hyper-aggressive fruit fly, *Drosophila prolongata*. Insect Biochem. Mol. Biol..

[11] Liang Q., Zhu B., Liu D., Lu Y., Zhang H., Wang F. (2023). Serotonin and dopamine regulate the aggressiveness of swimming crabs (*Portunus trituberculatus*) in different ways. Physiol. Behav..

[12] Momohara Y., Minami H., Kanai A., Nagayama T. (2016). Role of cAMP signalling in winner and loser effects in crayfish agonistic encounters. Eur. J. Neurosci..

[13] Yang X.-Z., Pang Y.-Y., Huang G.-Y., Xu M.-J., Zhang C., He L., Lv J.-H., Song Y.-M., Song X.-Z., Cheng Y.-X. (2019). The serotonin or dopamine by cyclic adenosine monophosphate-protein kinase A pathway involved in the agonistic behaviour of Chinese mitten crab, *Eriocheir sinensis*. Physiol. Behav..

[14] Ibuchi K., Nagayama T. (2021). Opposing effects of dopamine on agonistic behaviour in crayfish. J. Exp. Biol..

[15] Bortolato M., Godar S.C., Melis M., Soggiu A., Roncada P., Casu A., Flore G., Chen K., Frau R., Urbani A. (2012). NMDARs mediate the role of monoamine oxidase A in pathological aggression. J. Neurosci..

[16] Silva A.J., Paylor R., Wehner J.M., Tonegawa S. (1992). Impaired spatial learning in alpha-calcium-calmodulin kinase II mutant mice. Science.

[17] Zoicas I., Kornhuber J. (2019). The role of the N-Methyl-D-Aspartate receptors in social behavior in rodents. Int. J. Mol. Sci..

[18] Zhong W., Hutchinson T.E., Chebolu S., Darmani N.A. (2014). Serotonin 5-HT3 receptor-mediated vomiting occurs via the activation of Ca^2+^/CaMKII-dependent ERK1/2 signaling in the least shrew (*Cryptotis parva*). PLoS ONE.

[19] Cai Q., Chen X., Zhu S., Nicoll R.A., Zhang M. (2023). Differential roles of CaMKII isoforms in phase separation with NMDA receptors and in synaptic plasticity. Cell Rep..

[20] Sanhueza M., Lisman J. (2013). The CaMKII/NMDAR complex as a molecular memory. Mol. Brain.

[21] Withee J.R., Rehan S.M. (2017). Social aggression, experience, and brain gene expression in a subsocial bee. Integr. Comp. Biol..

[22] Hasegawa S., Furuichi T., Yoshida T., Endoh K., Kato K., Sado M., Maeda R., Kitamoto A., Miyao T., Suzuki R. (2009). Transgenic up-regulation of alpha-CaMKII in forebrain leads to increased anxiety-like behaviors and aggression. Mol. Brain.

[23] Chen C., Rainnie D.G., Greene R.W., Tonegawa S. (1994). Abnormal fear response and aggressive behavior in mutant mice deficient for α-calcium-calmodulin kinase, II. Science.

[24] Kim J.J., DeCola J.P., Landeira-Fernandez J., Fanselow M.S. (1991). N-methyl-D-aspartate receptor antagonist APV blocks acquisition but not expression of fear conditioning. Behav. Neurosci..

[25] Golden S.A., Heshmati M., Flanigan M., Christoffel D.J., Guise K., Pfau M.L., Aleyasin H., Menard C., Zhang H., Hodes G.E. (2016). Basal forebrain projections to the lateral habenula modulate aggression reward. Nature.

[26] Chiu H., Hoopfer E.D., Coughlan M.L., Pavlou H.J., Goodwin S.F., Anderson D.J. (2021). A circuit logic for sexually shared and dimorphic aggressive behaviors in *Drosophila*. Cell.

[27] Ishii K., Cortese M., Leng X.B., Shokhirev M.N., Asahina K. (2022). A neurogenetic mechanism of experience-dependent suppression of aggression. Sci. Adv..

[28] Sengupta S., Chan Y.B., Palavicino-Maggio C.B., Kravitz E.A. (2022). GABA transmission from mAL interneurons regulates aggression in *Drosophila* males. Proc. Natl. Acad. Sci. USA.

[29] Traniello I.M., Bukhari S.A., Dibaeinia P., Serrano G., Avalos A., Ahmed A.C., Sankey A.L., Hernaez M., Sinha S., Zhao S.D. (2023). Single-cell dissection of aggression in honeybee colonies. Nat. Ecol. Evol..

[30] Oliveira R.F., Simoes J.M., Teles M.C., Oliveira C.R., Becker J.D., Lopes J.S. (2016). Assessment of fight outcome is needed to activate socially driven transcriptional changes in the zebrafish brain. Proc. Natl. Acad. Sci. USA.

[31] Hepp Y., Tano M.C., Pedreira M.E., Freudenthal R.A. (2013). NMDA-like receptors in the nervous system of the crab *Neohelice granulata*: A neuroanatomical description. J. Comp. Neurol..

[32] Hepp Y., Salles A., Carbo-Tano M., Pedreira M.E., Freudenthal R. (2016). Surface expression of NMDA receptor changes during memory consolidation in the crab *Neohelice granulata*. Learn. Mem..

[33] Sandeman D., Sandeman R., Derby C., Schmidt M. (1992). Morphology of the brain of crayfish, crabs, and spiny lobsters: A common nomenclature for homologous structures. Biol. Bull..

[34] O’Connell L.A., Hofmann H.A. (2012). Evolution of a vertebrate social decision-making network. Science.

[35] Kravitz E.A. (1988). Hormonal control of behavior: Amines and the biasing of behavioral output in lobsters. Science.

[36] Northcutt A.J., Lett K.M., Garcia V.B., Diester C.M., Lane B.J., Marder E., Schulz D.J. (2016). Deep sequencing of transcriptomes from the nervous systems of two decapod crustaceans to characterize genes important for neural circuit function and modulation. BMC Genom..

[37] Sandeman D.C., Kenning M., Harzsch S. (2014). Adaptive trends in malacostracan brain form and function related to behavior. Crustacean Nervous System and Their Control Of Behaviour.

[38] Meth R., Wittfoth C., Harzsch S. (2017). Brain architecture of the Pacific White Shrimp *Penaeus vannamei* Boone, 1931 (Malacostraca, Dendrobranchiata): Correspondence of brain structure and sensory input?. Cell Tissue Res..

[39] Chen K. (1980). The anatomy of the central nerve system of *Penaeus orientalis* and *Portunus trituberculatus*, and comparative morphology of the nervous-Chain of decapoda. J. Shandong Coll. Oceanol..

[40] Khornchatri K., Kornthong N., Saetan J., Tinikul Y., Chotwiwatthanakun C., Cummins S.F., Hanna P.J., Sobhon P. (2015). Distribution of serotonin and dopamine in the central nervous system of the female mud crab, *Scylla olivacea* (Herbst). Acta Histochem..

[41] Certel S.J., Savella M.G., Schlegel D.C.F., Kravitz E.A. (2007). Modulation of *Drosophila* male behavioral choice. Proc. Natl. Acad. Sci. USA.

[42] Wine J.J., Krasne F.B. (1972). The organization of escape behaviour in the crayfish. J. Exp. Biol..

[43] Momohara Y., Aonuma H., Nagayama T. (2018). Tyraminergic modulation of agonistic outcomes in crayfish. J. Comp. Physiol. A.

[44] Sato N., Shidara H., Kamo S., Ogawa H. (2022). Roles of neural communication between the brain and thoracic ganglia in the selection and regulation of the cricket escape behavior. J. Insect Physiol..

[45] Young R.E., Govind C.K. (1983). Neural asymmetry in male fiddler crabs. Brain Res..

[46] Liang Q., Liu D., Zhang D., Wang X., Zhu B., Wang F. (2024). Machine learning-based aggressiveness assessment model construction for crabs: A case study of swimming crab *Portunus trituberculatus*. Aquaculture.

[47] Chen K., Yang L.N., Lai C., Liu D., Zhu L.Q. (2020). Role of Grina/Nmdara1 in the central nervous system diseases. Curr. Neuropharmacol..

[48] Incontro S., Diaz-Alonso J., Iafrati J., Vieira M., Asensio C.S., Sohal V.S., Roche K.W., Bender K.J., Nicoll R.A. (2018). The CaMKII/NMDA receptor complex controls hippocampal synaptic transmission by kinase-dependent and independent mechanisms. Nat. Commun..

[49] Kalinine E., Zimmer E.R., Zenki K.C., Kalinine I., Kazlauckas V., Haas C.B., Hansel G., Zimmer A.R., Souza D.O., Müller A.P. (2014). Nandrolone-induced aggressive behavior is associated with alterations in extracellular glutamate homeostasis in mice. Horm. Behav..

[50] Lischinsky J.E., Lin D. (2020). Neural mechanisms of aggression across species. Nat. Neurosci..

[51] Roberton T., Daffern M., Bucks R.S. (2012). Emotion regulation and aggression. Aggress. Violent Behav..

[52] Bacque-Cazenave J., Cattaert D., Delbecque J.P., Fossat P. (2018). Alteration of size perception: Serotonin has opposite effects on the aggressiveness of crayfish confronting either a smaller or a larger rival. J. Exp. Biol..

[53] Goldstein R.S., Camhi J.M. (1991). Different effects of the biogenic amines dopamine, serotonin and octopamine on the thoracic and abdominal portions of the escape circuit in the cockroach. J. Comp. Physiol. A.

[54] Bliss T.V.P., Collingridge G.L. (1993). A synaptic model of memory: Long-term potentiation in the hippocampus. Nature.

[55] Sanhueza M., Fernandez-Villalobos G., Stein I.S., Kasumova G., Zhang P., Bayer K.U., Otmakhov N., Hell J.W., Lisman J. (2011). Role of the CaMKII/NMDA receptor complex in the maintenance of synaptic strength. J. Neurosci..

[56] Abdoulaye I.A., Wu S.S., Chibaatar E., Yu D.F., Le K., Cao X.J., Guo Y.-J. (2021). Ketamine induces lasting antidepressant effects by modulating the NMDAR/CaMKII-mediated synaptic plasticity of the hippocampal dentate gyrus in depressive stroke model. Neural Plast..

[57] Baier A., Wittek B., Brembs B. (2002). *Drosophila* as a new model organism for the neurobiology of aggression?. J. Exp. Biol..

[58] Rollmann S.M., Zwarts L., Edwards A.C., Yamamoto A., Callaerts P., Norga K., Mackay T.F.C., Anholt R.R.H. (2008). Pleiotropic effects of Drosophila *neuralized* on complex behaviors and brain structure. Genetics.

[59] Edwards A.C., Zwarts L., Yamamoto A., Callaerts P., Mackay T.F. (2009). Mutations in many genes affect aggressive behavior in *Drosophila melanogaster*. BMC Biol..

[60] Zwarts L., Magwire M.M., Carbone M.A., Versteven M., Herteleer L., Anholt R.R., Callaerts P., Mackay T.F.C. (2011). Complex genetic architecture of *Drosophila* aggressive behavior. Proc. Natl. Acad. Sci. USA.

[61] Haddad J.J. (2005). N-methyl-D-aspartate (NMDA) and the regulation of mitogen-activated protein kinase (MAPK) signaling pathways: A revolving neurochemical axis for therapeutic intervention?. Prog. Neurobiol..

[62] Wang R., Reddy P.H. (2017). Role of glutamate and NMDA receptors in Alzheimer’s disease. J. Alzheimer’s Dis..

[63] Alaux C., Sinha S., Hasadsri L., Hunt G.J., Guzmán-Novoa E., DeGrandi-Hoffman G., Uribe-Rubio J.L., Southey B.R., Rodriguez-Zas S., Robinson G.E. (2009). Honey bee aggression supports a link between gene regulation and behavioral evolution. Proc. Natl. Acad. Sci. USA.

[64] Rittschof C.C., Vekaria H.J., Palmer J.H., Sullivan P.G. (2018). Brain mitochondrial bioenergetics change with rapid and prolonged shifts in aggression in the honey bee, *Apis mellifera*. J. Exp. Biol..

[65] Adamo S.A., Jensen M., Younger M. (2001). Changes in lifetime immunocompetence in male and female *Gryllus texensis* (formerly, G. *integer*): Trade-offs between immunity and reproduction. Anim. Behav..

[66] Rittschof C.C., Rubin B.E.R., Palmer J.H. (2019). The transcriptomic signature of low aggression in honey bees resembles a response to infection. BMC Genom..

[67] Camazine S. (1986). Differential reproduction of the mite, *Varroa jacobsoni* (Mesostigmata: Varroidae), on Africanized and European honey bees (Hymenoptera: Apidae). Ann. Entomol. Soc. Am..

[68] Rittschof C.C., Robinson G.E. (2013). Manipulation of colony environment modulates honey bee aggression and brain gene expression. Genes Brain Behav..

[69] Rittschof C.C., Vekaria H.J., Palmer J.H., Sullivan P.G. (2019). Biogenic amines and activity levels alter the neural energetic response to aggressive social cues in the honey bee *Apis mellifera*. J. Neurosci. Res..

[70] Salaciak K., Koszalka A., Zmudzka E., Pytka K. (2021). The calcium/calmodulin-dependent kinases II and IV as therapeutic targets in neurodegenerative and neuropsychiatric disorders. Int. J. Mol. Sci..

[71] Insel T.R., Young L.J. (2000). Neuropeptides and the evolution of social behavior. Curr. Opin. Neurobiol..

[72] Ball G.F., Balthazart J. (2004). Hormonal regulation of brain circuits mediating male sexual behavior in birds. Physiol. Behav..

[73] Yu Q., Teixeira C.M., Mahadevia D., Huang Y., Balsam D., Mann J.J., Gingrich J.A., Ansorge M.S. (2014). Dopamine and serotonin signaling during two sensitive developmental periods differentially impact adult aggressive and affective behaviors in mice. Mol. Psychiatry.

[74] Li-Byarlay H., Rittschof C.C., Massey J.H., Pittendrigh B.R., Robinson G.E. (2014). Socially responsive effects of brain oxidative metabolism on aggression. Proc. Natl. Acad. Sci. USA.

[75] Gomora-Garcia J.C., Montiel T., Huttenrauch M., Salcido-Gomez A., Garcia-Velazquez L., Ramiro-Cortes Y., Gomora J.C., Castro-Obregón S., Massieu L. (2023). Effect of the ketone body, D-beta-Hydroxybutyrate, on sirtuin2-mediated regulation of mitochondrial quality control and the autophagy-lysosomal pathway. Cells.

[76] Yan Y., Mukherjee S., Harikumar K.G., Strutzenberg T.S., Zhou X.E., Suino-Powell K., Xu T.-H., Sheldon R.D., Lamp J., Brunzelle J.S. (2021). Structure of an AMPK complex in an inactive, ATP-bound state. Science.

[77] Mandic M., Paunovic V., Vucicevic L., Kosic M., Mijatovic S., Trajkovic V., Harhaji-Trajkovic L. (2024). No energy, no autophagy-Mechanisms and therapeutic implications of autophagic response energy requirements. J. Cell. Physiol..

[78] Livak K.J., Schmittgen T.D. (2001). Analysis of relative gene expression data using real-time quantitative PCR and the 2(-Delta Delta C(T)) Method. Methods.

