# Supplementary material for: NMDAR-CaMKII Pathway as a Central Regulator of Aggressiveness: Evidence from Transcriptomic and Metabolomic Analysis in Swimming Crabs Portunus trituberculatus
Source: Int J Mol Sci. 2024 Nov 22;25(23):12560. doi: 10.3390/ijms252312560 (PMC11640798; doi:10.3390/ijms252312560)
Supplement: Supplementary file 1 [file ijms-25-12560-s001.zip › Supplementary Table S1, Figure S1-S5.pdf]

Table S1. Primer sequence

Gene	Primer sequence 5'-3'
NR2B	F: CATTGGCACGAGGTTTCGAC
	R: TCTGACCTGCCCTTGGTACT
CaMKII	F: AGGCTGACGCATCTCATTGTATTC
	R: GCCAACCCAAAGTCTGCTAGTTT
CREB	F: ATTCCTCACAAGACGAACCAACTG
	R: TGACCTGCCAACTGTAATCCACTA
ND1	F: AAGCAAGAAGCAAACCAAACCATTC
	R: TTGTTAGGAAGACTTCGGAGTGTTG
COX2	F: CGAGTAGTTATTACAGCAGCAGAT
	R: CTGAACATTGACCGTAGAATAATCC
CYTB	F: CTCCCGCTCATATTCAGCCAGA
	R: GCAACGGAAGCAACAAGAGCAA
ATP6	F: CCTCAAGGAACTCCACCTGTCTT
	R: TAGCAGCTAATCGAACAGCAAGTG
FOSLN	F: CGCCTCGACCAGACCAATATGC
	R: CCTTGTGTGTTGCCAGCAGAAATTC
FOXO	F: CGACGCAGAGCCAACACCAT
	R: CAGGTTCTCATTCAAGGACGAGGAG
SERCA	F: CGCTATTCCAGACCCCAAGG
	R: TCAGCCATCTGGGTACGGAT
PKA	F: CGTAAGAGTAAGGAGCTGGTGGT
	R: GGTTGGAGGAGGAGAGTTTAGGG
β-actin	F: CGAAACCTTCAACACTCCCG
	R: GATAGCGTGAGGAAGGGCATA

Note: NR2B: N-methyl-D-aspartate receptor 2B. CaMKII: calcium ion activation of calmodulin-dependent kinase II. CREB: cyclic AMP-responsive element-binding protein. ND1: NADH-ubiquinone oxidoreductase chain 1. COX2: cytochrome c oxidase subunit 2. CYTB: cytochrome b. ATP6: ATP synthase F0 subunit 6. FOSLN: AP-1 trancription factor subunit. FOXO: forkhead box protein O. SERCA: calcium-transporting ATPase sarcoplasmic/endoplasmic reticulum type. PKA: cAMP-dependent protein kinase catalytic subunit 1.

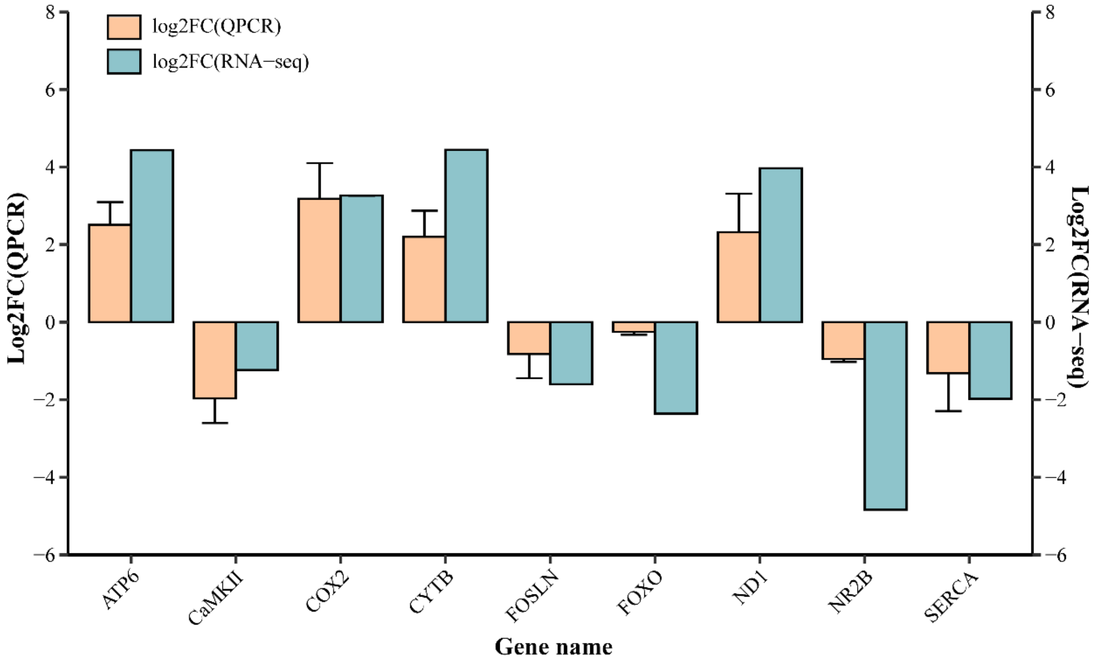

Figure S1. Relative expression of differential genes by transcriptome sequencing and qRT-PCR in the thoracic ganglion. The left Y axis represents the gene relative expression levels of qRT-PCR genes, and the right Y axis represents the gene relative expression levels of transcriptome. Positive value indicates a significant increase. Negative value indicates a significant decrease. Data are presented as the mean ± SD (n = 3).

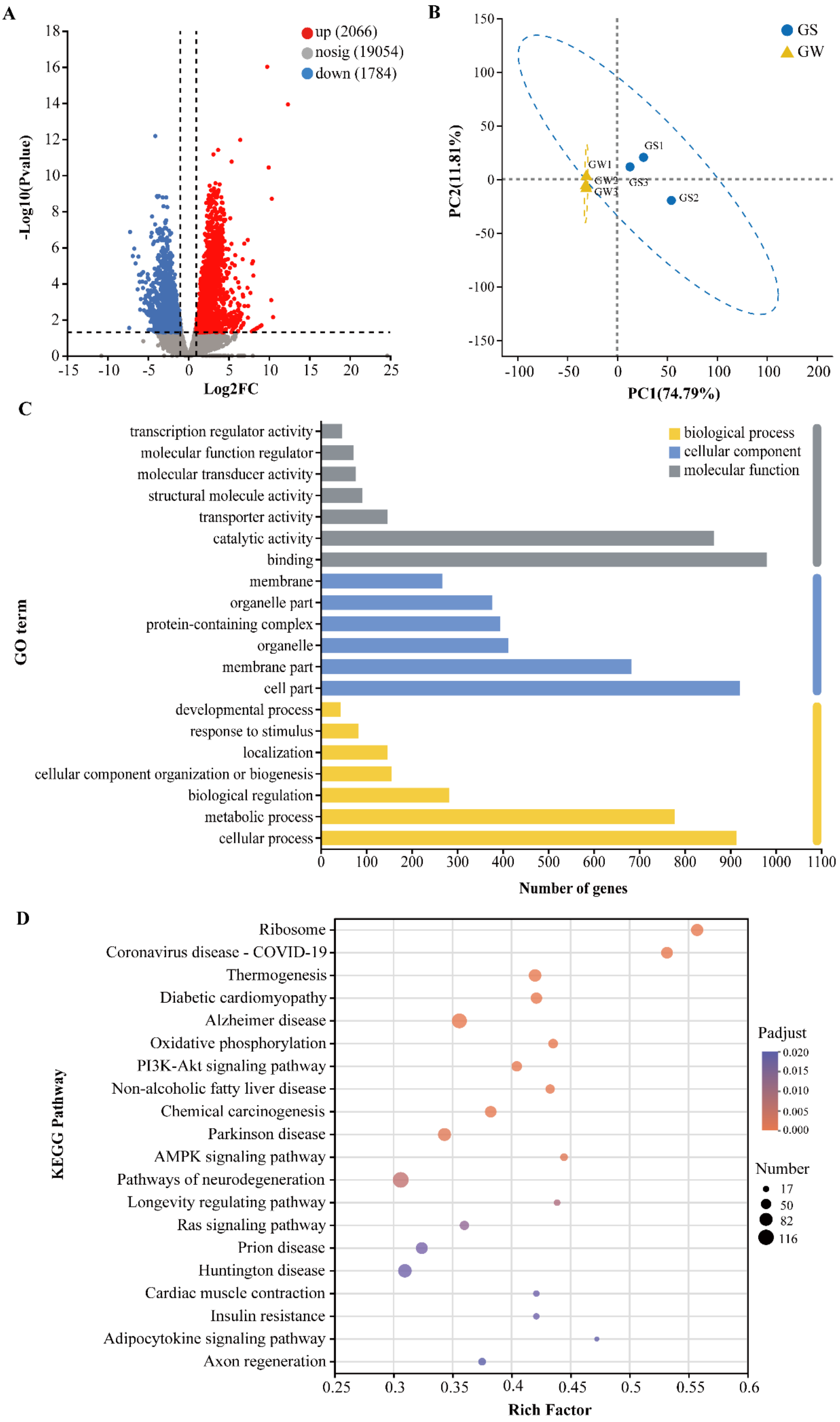

A
up (2066)
nosig (19054)
down (1784)
-Log10(Pvalue)
Log2FC
B
GS
GW
GS1
GS2
GS3
GW1
GW2
GW3
PC2(11.81%)
PC1(74.79%)
C
transcription regulator activity
molecular function regulator
molecular transducer activity
structural molecule activity
transporter activity
catalytic activity
binding
membrane
organelle part
protein-containing complex
organelle
membrane part
cell part
developmental process
response to stimulus
localization
cellular component organization or biogenesis
biological regulation
metabolic process
cellular process
biological process
cellular component
molecular function
GO term
Number of genes
D
Ribosome
Coronavirus disease - COVID-19
Thermogenesis
Diabetic cardiomyopathy
Alzheimer disease
Oxidative phosphorylation
PI3K-Akt signaling pathway
Non-alcoholic fatty liver disease
Chemical carcinogenesis
Parkinson disease
AMPK signaling pathway
Pathways of neurodegeneration
Longevity regulating pathway
Ras signaling pathway
Prion disease
Huntington disease
Cardiac muscle contraction
Insulin resistance
Adipocytokine signaling pathway
Axon regeneration
KEGG Pathway
Rich Factor
Padjust
Number

Figure S2. Transcriptome analysis of thoracic ganglia in male *Portunus trituberculatus* exhibiting strong and weak aggressiveness. (n = 3) (A) Volcano plot showing gene expression differences between strong and weak aggression. Red indicates significantly upregulated genes, blue indicates significantly downregulated genes, and gray indicates non-significant differences. (B) PCA analysis of the samples. (C) GO functional annotation of differentially expressed genes (DEGs). The y-axis shows GO secondary classification terms, while the x-axis displays the number of genes/transcripts mapped to these classifications. (D) KEGG Pathway enrichment analysis of DEGs. The y-axis shows pathway names, and the x-axis represents the rich factor (the ratio of enriched genes to annotated genes in the background). A higher rich factor indicates greater enrichment. The top 20 enrichment results with *P-adjust* <1 are displayed.

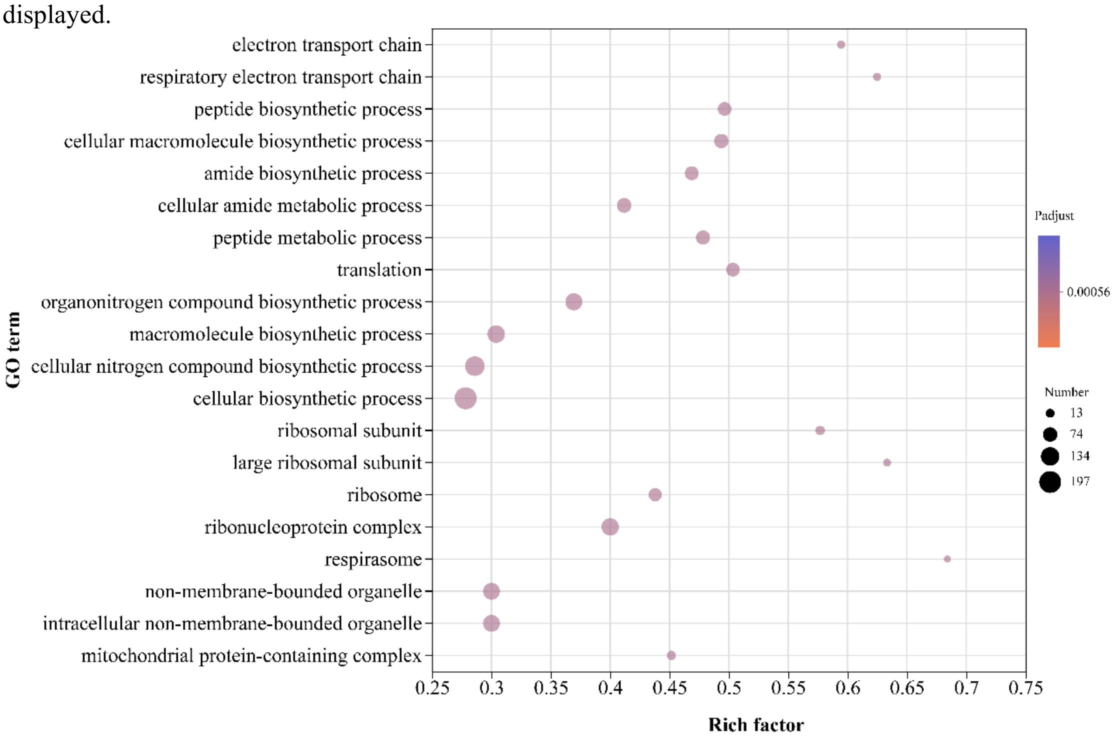

Figure S3. GO enrichment analysis of differentially expressed genes.

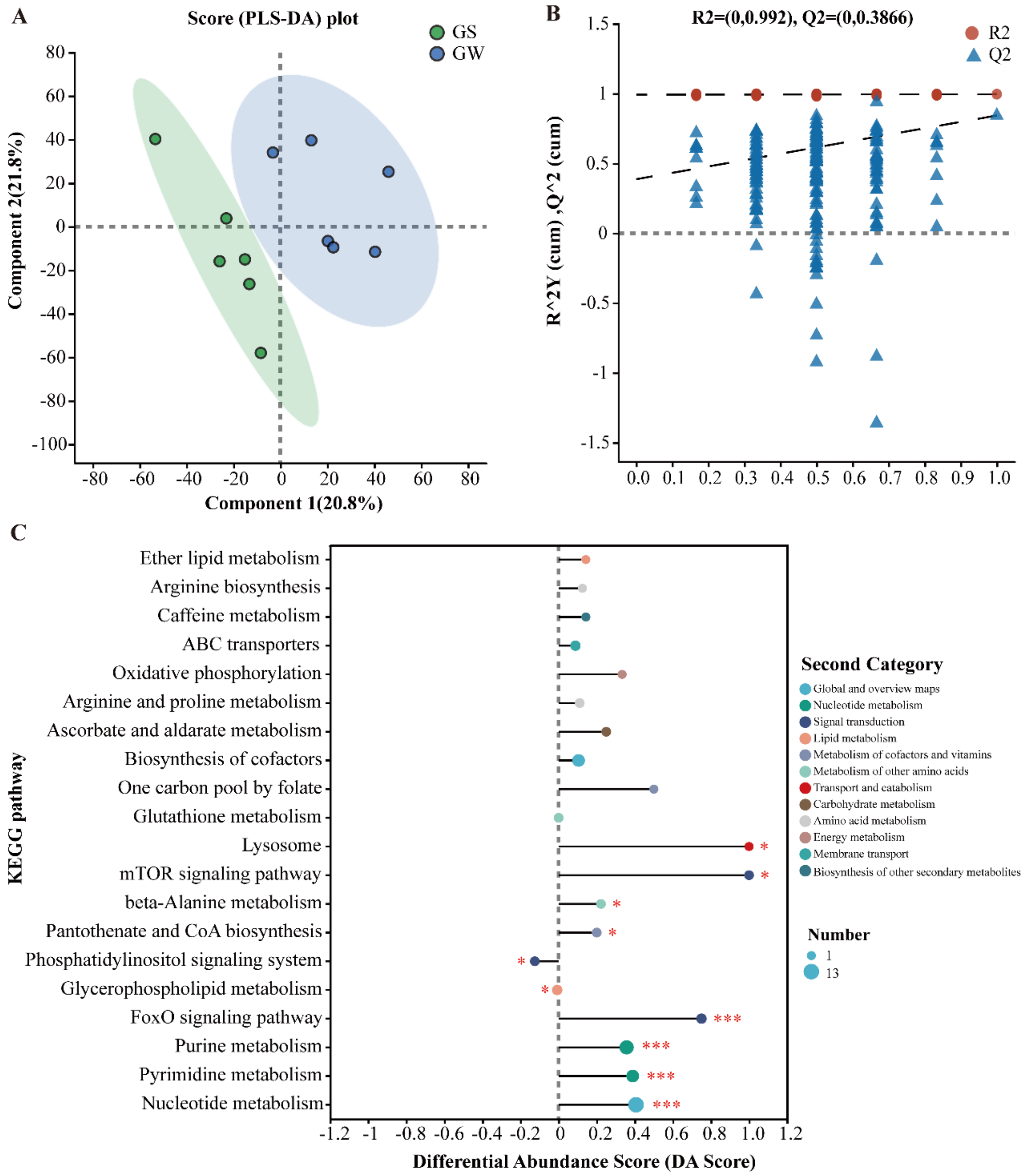

Figure S4. Metabolomic differences in the thoracic ganglia of male *Portunus trituberculatus* with varying levels of aggressiveness. (n = 6) (A) PLS-DA score plot. The horizontal axis represents the variance explained by the first principal component, while the vertical axis represents that of the second component. Greater separation indicates more significant classification. (B) PLS-DA model validation. The horizontal axis represents permutation retention, while the vertical axis shows R^2 (red circles) and Q^2 (blue triangles) values. Dashed lines represent regression lines for R^2 and Q^2. The model was validated with 200 random permutations. (C) KEGG pathway differential abundance score plot. The horizontal axis shows the differential abundance score, while the vertical axis lists KEGG pathways. A DA score of “1” indicates an uptrend in all annotated metabolites, and “-1” indicates a downtrend. Line length represents the absolute DA score. Dot size corresponds to the number of annotated differential metabolites, with larger dots indicating more metabolites.

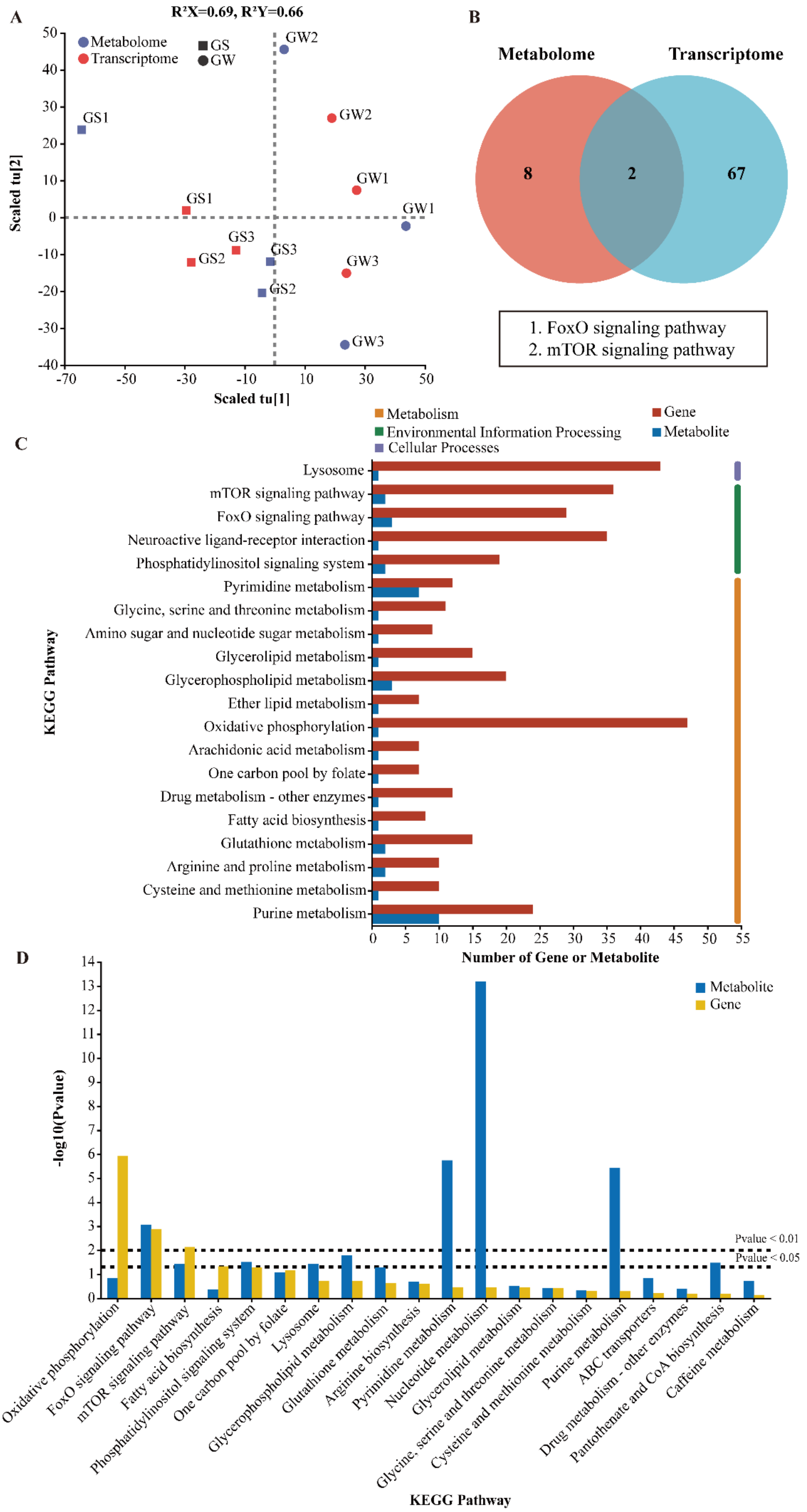
A
R²X=0.69, R²Y=0.66
Metabolome
Transcriptome
GS
GW
GS1
GS2
GS3
GW1
GW2
GW3
Scaled tu[2]
Scaled tu[1]
B
Metabolome
Transcriptome
8
2
67
1. FoxO signaling pathway
2. mTOR signaling pathway
C
Metabolism
Environmental Information Processing
Cellular Processes
Gene
Metabolite
Lysosome
mTOR signaling pathway
FoxO signaling pathway
Neuroactive ligand-receptor interaction
Phosphatidylinositol signaling system
Pyrimidine metabolism
Glycine, serine and threonine metabolism
Amino sugar and nucleotide sugar metabolism
Glycerolipid metabolism
Glycerophospholipid metabolism
Ether lipid metabolism
Oxidative phosphorylation
Arachidonic acid metabolism
One carbon pool by folate
Drug metabolism - other enzymes
Fatty acid biosynthesis
Glutathione metabolism
Arginine and proline metabolism
Cysteine and methionine metabolism
Purine metabolism
KEGG Pathway
Number of Gene or Metabolite
D
Metabolite
Gene
-log10(Pvalue)
Pvalue < 0.01
Pvalue < 0.05
Oxidative phosphorylation
FoxO signaling pathway
mTOR signaling pathway
Fatty acid biosynthesis
Phosphatidylinositol signaling system
One carbon pool by folate
Lysosome
Glycerophospholipid metabolism
Glutathione metabolism
Arginine biosynthesis
Pyrimidine metabolism
Nucleotide metabolism
Glycerolipid metabolism
Glycine, serine and threonine metabolism
Cysteine and methionine metabolism
Purine metabolism
ABC transporters
Drug metabolism - other enzymes
Pantothenate and CoA biosynthesis
Caffeine metabolism
KEGG Pathway

Figure S5. Integrated analysis of transcriptomics and metabolomics results. (A) O2PLS analysis results. Point shapes represent different sample groups, and point colors represent different omics samples. The horizontal and vertical axes show combined scores from metabolomics and transcriptomics, with "t" denoting transcriptomics scores and "u" representing metabolomics scores. R^2X reflects the proportion of variation in the X matrix related to modeling (joint and orthogonal variation) relative to the total X matrix variation. Similarly, R^2Y reflects the proportion of variation in the Y matrix related to modeling relative to the total Y matrix variation. Higher R^2X and R^2Y values indicate better model association. (B) Venn diagram of KEGG pathway enrichment. (C) KEGG annotation statistics. The horizontal axis shows the number of genes or metabolites in each pathway, while the vertical axis lists the top 20 KEGG-annotated pathways for differential genes and metabolites. Blue bars represent the number of genes, red bars represent the number of metabolites, and yellow, green, and purple bars indicate the primary classification names of the enriched pathways. (D) KEGG pathway enrichment plot. The horizontal axis lists the top 20 pathways enriched by differential genes and metabolites, with yellow representing gene enrichment and blue representing metabolite annotation. The vertical axis shows the negative logarithm (base 10) of the enrichment significance (P-value). Smaller p-values indicate higher statistical significance, with $P < 0.05$ generally considered significant for pathway enrichment.
